# Childhood systemic glucocorticoid exposure and subsequent alterations in myocardial structure and cardiac function: a narrative review of clinical evidence

**DOI:** 10.3389/fendo.2026.1781454

**Published:** 2026-03-26

**Authors:** Wenxuan Li, Yangang Wang

**Affiliations:** The Affiliated Hospital of Qingdao University, Qingdao, China

**Keywords:** cardiac remodeling, cardiovascular outcomes, pediatrics, subclinical cardiac dysfunction, systemic glucocorticoids

## Abstract

**Background:**

Systemic glucocorticoids (GCs) remain indispensable in pediatric care, yet their potential long-term cardiovascular sequelae—particularly after exposure during developmental windows—are incompletely defined.

**Objective:**

To synthesize representative clinical evidence linking childhood/adolescent systemic GC exposure with cardiac structure remodeling, subclinical functional alterations, and longer-term cardiovascular outcomes, and to contextualize plausibly mediating pathophysiologic pathways.

**Evidence synthesis:**

Across pediatric conditions with repeated or prolonged systemic GC use, the most consistent structural signal is a time-locked, reversible myocardial hypertrophy phenotype in neonates/young infants—often observed during or shortly after dexamethasone exposure and regressing after dose reduction or discontinuation. In older children and adolescents, evidence for persistent, overt structural damage is sparse and confounded by underlying disease and treatment context. Conventional systolic indices (e.g., EF) are frequently preserved, while more sensitive metrics (tissue Doppler–derived parameters, myocardial performance index, strain) can reveal mild, subclinical systolic–diastolic impairment in selected populations (e.g., congenital adrenal hyperplasia). For “hard” adult outcomes (heart failure, coronary events, atrial fibrillation), direct longitudinal pediatric-to-adult data remain limited; however, pediatric GC exposure shows clearer dose–time associations with intermediate cardiometabolic risk factors and thromboembolic events, supporting a plausible mediated pathway to later cardiovascular disease.

**Conclusions:**

Current pediatric evidence more strongly supports transient remodeling and risk-factor clustering than definitive, irreversible cardiomyopathy. Future studies need long-horizon, indication-aware cohorts with harmonized imaging and event endpoints to quantify exposure–response relationships and identify actionable mediators.

## 1 Introduction

Glucocorticoids are among the most frequently prescribed—and arguably least replaceable—agents in pediatric practice, being routinely used for both induction and maintenance therapy across a broad spectrum of conditions, including asthma, allergic disorders, autoimmune diseases, nephrotic syndrome, acute leukemia, and severe infections. In many children, exposure extends throughout childhood and even into adolescence, constituting a cornerstone of disease control strategies (1). Yet, as treatment duration lengthens and cumulative dose accrues, systemic adverse effects have become increasingly prominent, ranging from weight gain, growth retardation, and Cushingoid features to osteoporosis, heightened susceptibility to infection, and disturbances in glucose and lipid metabolism (1, 2).

Systemic glucocorticoids (GCs) are steroid hormones and pharmacologic analogues that exert pleiotropic effects primarily through the glucocorticoid receptor (GR), a ligand-activated transcription factor expressed across immune cells, vascular tissues, and the myocardium. Their clinical utility in pediatrics derives largely from potent anti-inflammatory and immunomodulatory actions, including suppression of pro-inflammatory transcriptional programs and downstream cytokine signaling, as well as modulation of leukocyte trafficking and endothelial activation. At the cardiovascular level, GCs can influence hemodynamics and metabolic homeostasis—through effects on sodium handling, vascular tone, sympathetic/RAAS signaling, and glucose–lipid metabolism—thereby shaping afterload and myocardial energy demand. In parallel, GR signaling in cardiomyocytes and vascular cells may directly regulate stress responses, growth/remodeling pathways, and extracellular matrix turnover. These actions can plausibly yield context-dependent effects: in some disease settings GCs may attenuate inflammatory injury and slow adverse remodeling, whereas in others prolonged exposure may amplify hypertension, dysmetabolism, prothrombotic tendencies, and remodeling substrates that contribute to long-term cardiovascular risk (1, 2).

In adults, the association between systemic glucocorticoid therapy and cardiovascular disease is supported by relatively robust epidemiological evidence. A case–control analysis drawing on a UK primary care database reported that individuals prescribed glucocorticoids had a significantly higher subsequent risk of cardiovascular events—including myocardial infarction, stroke, and heart failure—and this relationship persisted even after adjustment for conventional risk factors (3). More recently, a large cohort study spanning six immune-mediated disorders and nearly 88,000 patients further demonstrated that even low-dose oral glucocorticoids (<5 mg prednisolone-equivalent/day) were linked to a meaningful increase in cardiovascular event risk, with a clear dose–response pattern: higher daily doses and greater cumulative exposure translated into progressively higher cardiovascular risk (4). Consistently, This focused clinical reviews and meta-analyses suggest that glucocorticoid use is associated with elevated risks of major adverse cardiovascular events (MACE), coronary heart disease, and heart failure, with risk rising in parallel with both daily and cumulative dose (5).

By contrast, the evidence base in children and adolescents remains far more fragmented. On the one hand, systemic glucocorticoids continue to be standard-of-care for many severe and chronic pediatric conditions: children with classic congenital adrenal hyperplasia (CAH) often receive lifelong glucocorticoid replacement from infancy, while those with nephrotic syndrome, systemic lupus erythematosus, or juvenile idiopathic arthritis frequently require recurrent or prolonged courses. On the other hand, much of the pediatric literature has centered on adverse effects such as impaired growth, skeletal toxicity, and infection risk, with comparatively limited attention to the long-term consequences for myocardial structure and cardiac performance (1, 2). Notably, a small number of studies in CAH and related populations suggest that clustering of cardiometabolic risk factors—including obesity, hypertension, insulin resistance, increased carotid intima–media thickness, and subclinical impairment of cardiac function—may already be evident during childhood and adolescence, raising concern that cardiovascular disease risk could be amplified later in adult life (6).

Conversely, some studies suggest that in specific disease contexts—most notably Duchenne muscular dystrophy—long-term glucocorticoid therapy may confer a degree of cardioprotection, delaying the onset of dilated cardiomyopathy and slowing the trajectory of functional deterioration. This observation underscores that the “glucocorticoid–heart” relationship is not uniformly deleterious; rather, it reflects a nuanced equilibrium shaped by the underlying disease milieu, dose intensity, and the duration and timing of exposure. Such a “double-edged sword” profile places clinicians in a familiar therapeutic dilemma when planning long-term treatment for children: ensuring sufficient glucocorticoid exposure to control the primary disorder while remaining vigilant about the possibility of latent cardiovascular toxicity.

To date, there remains no comprehensive synthesis that systematically addresses several clinically pivotal questions: whether systemic glucocorticoid exposure during childhood or adolescence increases the risk of subsequent abnormalities in myocardial structure and cardiac function—particularly diastolic performance and longitudinal mechanics; whether such changes translate into clinically overt outcomes such as heart failure, arrhythmias, or other adverse cardiovascular events; whether risk follows a dose–time gradient rather than representing an inevitable consequence of exposure; and whether the long-term risk profile differs between developmental exposure and treatment initiated in adulthood.

**Figure 1:**
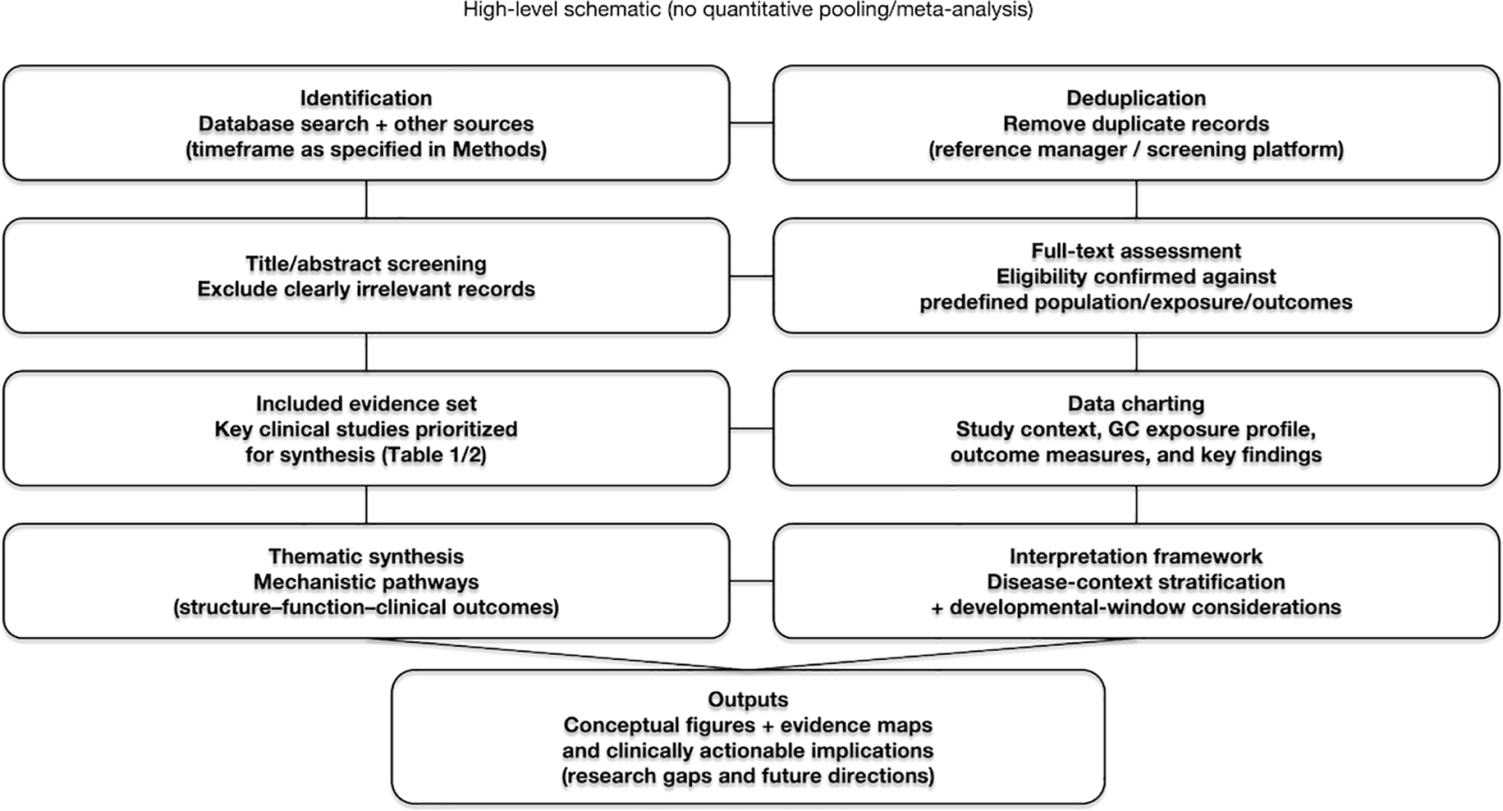
Review workflow and evidence synthesis schematic.

Against this background, the present review consolidates the available clinical literature and key lines of evidence on the cardiovascular sequelae of systemic glucocorticoid exposure in children and adolescents. We focus on whether exposure is linked to myocardial remodeling and subclinical functional impairment; the extent to which current data support longer-term outcomes such as heart failure and arrhythmias; whether adverse cardiac signals exhibit dose-, duration-, or cumulative exposure–related trends; and, informed by relevant basic and translational insights, the plausibly mediating pathophysiological pathways—thereby providing a more actionable framework for risk identification, monitoring, and follow-up in children requiring prolonged glucocorticoid therapy. To make this synthesis transparent and clinically actionable, we summarize our evidence-identification and synthesis workflow (**Figure 1**) and then organize the discussion along a structure–function–clinical outcomes continuum, stratified by disease context and developmental timing.

## 2 Methods

This article is a narrative, clinically focused review that synthesizes representative clinical studies on the cardiovascular consequences of systemic glucocorticoid exposure during childhood and adolescence. Evidence is organized along a structure–function–clinical outcomes framework and stratified by underlying disease context, with emphasis on exposure intensity (dose, duration, cumulative burden), developmental timing, and sources of heterogeneity and confounding. Given the variability in exposure definitions, follow-up windows, and outcome ascertainment across available studies, this review is presented as a qualitative synthesis rather than a quantitative meta-analysis (7, 8).

Focusing on pediatric and adolescent systemic glucocorticoid exposure, we examine its associations with cardiac structural remodeling, functional alterations, and clinical cardiovascular outcomes by surveying key studies and relevant reviews across diverse disease contexts, and by organizing evidence thematically according to major drug classes and outcome domains (structure, function, and clinical events) (9).

The discussion is structured around three outcome categories—myocardial structure (e.g., left ventricular mass, wall thickness, and remodeling), cardiac function (e.g., ejection fraction, strain, and diastolic indices), and clinical endpoints (e.g., heart failure, arrhythmias, and major adverse cardiovascular events). To enhance interpretability, we align, where feasible, exposure definitions (drug type, route of administration, duration, and cumulative dose) with outcome assessment modalities (conventional echocardiography, tissue Doppler, strain imaging, and related techniques), and emphasize disease-stratified interpretation to reduce conceptual ambiguity and improve cross-study comparability (7).

Given substantial heterogeneity in study design, exposure characterization, follow-up duration, and outcome measurement—along with pervasive confounding—the interpretation of individual findings is explicitly contextualized by methodological limitations and potential sources of bias, avoiding undue extrapolation of associative evidence to causal inference (10–12).

Evidence is further stratified by underlying disease category (e.g., immune-mediated disorders, kidney diseases, endocrine conditions, and neuromuscular diseases) and synthesized along a “structure–function–clinical outcomes” continuum. Within each category, we highlight whether a consistent directionality and biologically plausible trend emerges with respect to exposure intensity (dose, duration, and cumulative burden). Because of marked between-study differences in population characteristics, exposure definitions, follow-up windows, and outcome ascertainment, this review is presented primarily as a qualitative synthesis with comparative discussion—emphasizing convergence of evidence and sources of discordance—rather than statistical pooling or quantitative inference (13, 14).A high-level schematic of the review workflow and evidence-synthesis approach is provided in **Figure 1** to guide readers through how the subsequent sections are organized and interpreted.

### 2.1 Evidence base and interpretive approach

This review examines the cardiovascular consequences of systemic glucocorticoid exposure during childhood and adolescence by synthesizing representative clinical studies across diverse disease contexts. Evidence is organized along a “structure–function–clinical outcomes” continuum, with particular attention to whether exposure intensity (dose, treatment duration, and cumulative burden) aligns with outcome changes in a consistent direction and with an interpretable trend. To help readers rapidly grasp the overall landscape, **Supplementary Table 1** summarizes the core studies, including population context, exposure characteristics, and principal outcome measures.

Several structural constraints, however, limit comparability across studies and restrict external generalizability. Marked differences exist in underlying disease spectra, treatment regimens and exposure characterization (including whether peak and/or cumulative doses are reported), follow-up duration, and cardiac assessment modalities (conventional echocardiography, tissue Doppler, strain imaging, and MRI or nuclear techniques). Moreover, because systemic glucocorticoids are typically prescribed for severe or chronic conditions, confounding by indication and disease-severity–related factors are difficult to fully eliminate. In light of these limitations, we adhere to disease-stratified interpretation throughout, and we assign more cautious weight to findings from studies with limited confounding control, imprecise exposure definitions, or non-comparable outcome measurements, thereby avoiding overextension of associative evidence into causal conclusions.

### 2.2 Mechanisms by which glucocorticoids influence the cardiovascular system

**Figure 2:**
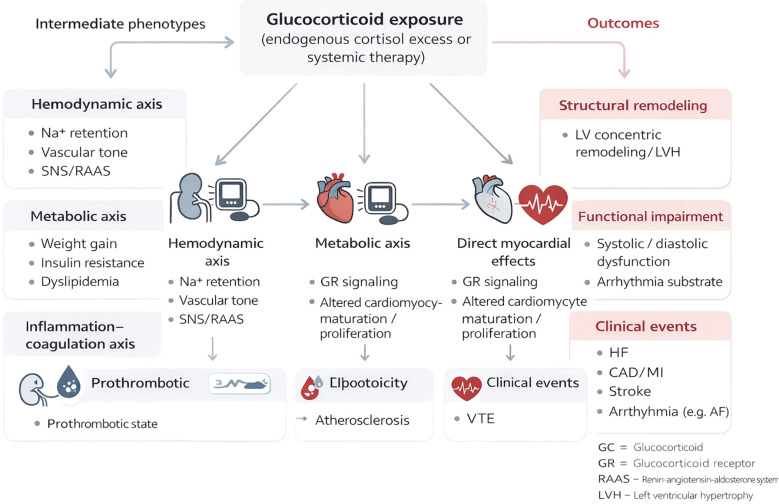
Pathways linking cortisol (glucocorticoid) exposure to cardiac and vascular outcomes.

Glucocorticoid (GC) effects on the cardiovascular system are best understood as a coordinated “load–remodeling–compensation/decompensation” process initiated across the kidney, vascular wall, and myocardium via the glucocorticoid receptor (GR), with additional crosstalk involving mineralocorticoid pathways (15, 16). Accordingly, clinical phenotypes such as left ventricular hypertrophy, impaired diastolic function, and an increased propensity for arrhythmias rarely reflect a single dominant mechanism; rather, they emerge from the cumulative impact of multiple converging axes (**Figure 2**) (16, 17).

#### 2.2.1 Hemodynamic and metabolic axis

Increased load first, remodeling thereafter. Exogenous GC exposure can induce or exacerbate hypertension through sodium and water retention, heightened vascular reactivity, and sympathetic/RAAS-related alterations (15). In parallel, GCs promote or amplify insulin resistance, hyperglycemia, dyslipidemia, and fat redistribution (16). Together, these changes sustain afterload and myocardial oxygen demand, favoring concentric remodeling and hypertrophy and creating a permissive substrate for subsequent diastolic impairment (e.g., pressure load–related reductions in compliance) (15, 16). Consistent with this framework, large-scale population studies report dose-related associations between systemic GC exposure and multiple cardiovascular outcomes, including heart failure and atrial fibrillation, supporting a real-world “cumulative exposure–event risk” gradient (3, 4).

#### 2.2.2 Direct myocardial effects

GR-mediated developmental programming and pro-fibrotic tendencies. Beyond indirect influences via blood pressure and metabolism, GCs may act directly on the myocardium. GR is widely expressed in cardiomyocytes and vascular endothelial/smooth muscle cells and regulates pathways governing cellular growth, stress responses, inflammation, and remodeling (19). Of particular relevance to pediatric exposure is a potential “programming effect” during developmental windows (fetal, neonatal, and early childhood). Experimental data suggest that early-life GC exposure can suppress physiologic cardiomyocyte proliferation and mitosis, accelerate terminal differentiation, and thereby increase vulnerability in later life to compensatory hypertrophy, impaired systolic/diastolic performance, or reduced stress reserve (18, 19). Related models have also linked early GC exposure to adult abnormalities in cardiac structure/function, shifts in cardiomyocyte number or phenotype, and fibrosis-related changes, providing biological plausibility for the concept that GC use during childhood/adolescence may leave a durable structural–functional imprint (18–21).

#### 2.2.3 Pediatric Cushing syndrome

A human “hypercortisolism model” demonstrating early cardiovascular remodeling. Pediatric Cushing syndrome (endogenous hypercortisolism) offers a clinically informative human analogue for sustained high cortisol exposure. Available studies indicate that affected children frequently develop hypertension and metabolic disturbances and may exhibit early vascular functional/structural signals, including increases in indices related to arterial stiffness (22, 23). Moreover, coagulation and fibrinolytic abnormalities consistent with hypercortisolism have been reported, suggesting that vascular event risk may reflect not only traditional cardiometabolic factors but also longer-term disruption along a “vessel wall–coagulation” axis (24). Collectively, these observations support the notion that persistent hypercortisol states—whether endogenous or iatrogenic—can produce measurable cardiovascular remodeling in pediatric populations (22–24).

#### 2.2.4 Inflammation–coagulation axis

Chronic hypercortisolism as a prothrombotic milieu. While GCs suppress inflammation in the short term, Cushing-related studies show that sustained or high exposure can be accompanied by upregulation of procoagulant factors, impaired fibrinolysis, and an increased thrombotic tendency (25). This focused clinical reviews similarly describe elevated venous thromboembolism risk in Cushing syndrome alongside a characteristic coagulation/fibrinolysis abnormality profile (25). In pediatric cohorts, prospective work has documented increases in several procoagulant and antifibrinolytic markers that partially regress after surgical remission, implying that the prothrombotic phenotype is at least partly reversible but may also exert lingering effects (24). This axis provides a mechanistic bridge to clinically meaningful endpoints such as thrombosis, stroke, and ischemic events (24, 25).

#### 2.2.5 A biphasic “protection–injury” paradigm governed by dose, timing, and host context

GC effects on the cardiovascular system are plausibly biphasic: physiologic levels contribute to homeostasis and stress adaptation, whereas excessive or prolonged exposure is more likely to drive adverse remodeling and event risk through the combined actions of hemodynamic, metabolic, direct myocardial, and coagulation pathways (16, 17). Thus, if future clinical studies confirm associations between pediatric GC exposure and later-life hypertrophy, diastolic impairment, heart failure, or arrhythmias, the most defensible interpretation will rarely be inevitability; more often, risk will reflect cumulative dose, duration, the developmental timing of exposure, and the overlay of underlying disease and coexisting risk factors (3, 4). This mechanistic framework also provides the rationale for stratifying evidence by dose/duration, disease context, follow-up horizon, and outcome modality in subsequent sections.

Building on this mechanistic framework, the following sections summarize how these pathways manifest clinically across developmental windows and disease contexts, first in cardiac structure and then in functional phenotypes and longer-term outcomes.

### 2.3 Effects of childhood glucocorticoid use on cardiac structure

At present, population-level evidence linking childhood/adolescent glucocorticoid exposure to persistent myocardial hypertrophy or fibrosis remains limited. The most consistent signal comes from systemic steroid exposure in the neonatal/infant period—particularly dexamethasone—typically presenting as time-dependent and reversible myocardial hypertrophy or a hypertrophic cardiomyopathy–like phenotype (26–29). Key clinical studies and their principal structural findings across disease contexts are summarized in **Supplementary Table 2**.

#### 2.3.1 Neonatal and infant period

The most consistent evidence—predominantly “transient, reversible hypertrophy.” In preterm infants treated with systemic dexamethasone for bronchopulmonary dysplasia (BPD) and related indications, echocardiography commonly demonstrates increased interventricular septal and left ventricular posterior wall thickness; in some cases, outflow tract obstruction or a hypertrophic cardiomyopathy–like presentation is observed. With dose tapering or discontinuation, these changes usually regress over weeks, suggesting heightened myocardial sensitivity to glucocorticoids in early life and a largely reversible structural response (26–28). Randomized controlled trials have similarly reported a higher likelihood of septal/posterior wall thickening in early dexamethasone groups versus controls, with a clear temporal relationship to treatment exposure (29).

#### 2.3.2 Childhood/adolescence

Sparse clinical evidence—mostly case reports/small series with substantial confounding. Beyond infancy, reports of “systemic glucocorticoids → definite myocardial hypertrophy” become noticeably less frequent and are largely confined to exceptional scenarios such as short-course, high-dose pulse therapy. For example, a case describing transient global ventricular hypertrophy during treatment for MIS-C, improving after dose reduction or discontinuation, suggests that in the setting of intense inflammation and physiological stress, high-dose glucocorticoids may coexist with reversible hypertrophy. However, given the profound influence of underlying disease severity and concomitant therapies, robust causal attribution is difficult (30).

#### 2.3.3 A special model of long-term/chronic exposure

Congenital adrenal hyperplasia (CAH) as informative indirect evidence. Because many children with CAH require prolonged glucocorticoid replacement, this population provides a clinically relevant model for chronic exposure. Case–control data in children with 21-hydroxylase deficiency generally show no definitive structural abnormalities such as left ventricular hypertrophy or chamber dilatation on echocardiography. While some parameters have been interpreted as suggesting mild functional changes, overall structural indices largely remain within the normal range (31).

#### 2.3.4 Longer-term follow-up

No clear persistent structural abnormalities at school age, but late effects remain uncertain. Follow-up studies of perinatal/neonatal glucocorticoid exposure into school age (approximately 7–10 years) have not demonstrated significant differences between exposed and unexposed groups in cardiac structure or routine systolic/diastolic measures. Importantly, these studies also emphasize that certain cardiovascular risks may emerge later in life, supporting continued follow-up to evaluate potential delayed effects (32).

Several non-mutually exclusive mechanisms may explain why structural signals appear more consistent in neonates/infants than in older children. Early life is characterized by higher myocardial plasticity, ongoing cardiomyocyte maturation, and rapid changes in loading conditions, potentially making the myocardium more responsive to exogenous steroid signaling. Pubertal transitions add further complexity by altering endogenous sex-steroid and growth hormone/IGF-1 axes, which can interact with cardiometabolic risk profiles and remodeling pathways. Moreover, GR expression, downstream co-regulator availability, and tissue sensitivity to glucocorticoids may be developmentally regulated, such that the same pharmacologic exposure yields distinct effects depending on the timing and the host disease milieu. These considerations support interpreting pediatric GC–cardiac associations through a “developmental window + disease context” framework rather than assuming uniform toxicity.

#### 2.3.5 Overall synthesis

Current clinical evidence most strongly supports a time-linked, reversible myocardial hypertrophy signal following high-dose systemic glucocorticoids in the neonatal/infant period (26–29). In childhood/adolescence, structural changes are primarily suggested by high-dose, context-specific case reports, where causal inference is constrained by confounding (30). In chronic replacement settings such as CAH, most studies do not show clear left ventricular hypertrophy or chamber enlargement, implying that “long-term exposure leading to fixed hypertrophy” is not an inevitable outcome (31). The dominant evidence gap remains well-powered, long-term, rigorously adjusted population studies using harmonized structural endpoints (e.g., LVMI, wall thickness Z-scores, and CMR-based fibrosis metrics) (32).

### 2.4 Effects of glucocorticoids on cardiac function in children and adolescents

#### 2.4.1 Systolic function

Most studies suggest that clinically overt systolic impairment is uncommon, but special contexts warrant caution. In follow-up cohorts of preterm infants treated with systemic glucocorticoids during the perinatal/infant period, conventional echocardiographic indices of systolic performance at school age (e.g., ejection fraction [EF] and fractional shortening) are generally preserved and do not differ significantly from those of unexposed controls (32). Small-sample longitudinal follow-up further indicates that even when early treatment is accompanied by steroid-associated transient hypertrophic cardiomyopathy (HCM) that subsequently resolves, systolic and diastolic function between 3 and 8 years of age remains overall reassuringly normal (33). Importantly, in the acute phase, a subset of preterm infants receiving an early, short-course dexamethasone regimen may develop left ventricular hypertrophy, sometimes with symptoms or hemodynamic consequences (29); this pattern is more consistent with a treatment-related, short-term reversible change than with an inevitable trajectory toward sustained long-term systolic dysfunction.

#### 2.4.2 Diastolic and subclinical function

Tissue Doppler–based and integrative indices appear more sensitive, suggesting that mild, subclinical changes may exist. This does not contradict the observation of “normal EF,” because differences may emerge only when more sensitive functional parameters are examined. In congenital adrenal hyperplasia (CAH), a clinical scenario characterized by long-term glucocorticoid replacement, a case–control study in Cardiology in the Young reported not only higher structural parameters in the CAH group (e.g., septal/posterior wall thickness and left ventricular mass index), but also significant differences in pulsed-wave and tissue Doppler–derived measures—such as isovolumic contraction/relaxation times and the myocardial performance index (MPI/Tei)—reflecting global myocardial performance (34). After accounting for hyperandrogenism, the average hydrocortisone dose was positively correlated with isovolumic relaxation time, supporting a signal consistent with dose-related functional adaptation or impairment (34). A Clinical Endocrinology (Oxf) study likewise suggested subtle left ventricular diastolic abnormalities in CAH (e.g., shortened isovolumic relaxation time interpreted as a marker of mild diastolic dysfunction), although overall function largely remained within conventional normal ranges (31). Another Clinical Endocrinology (Oxf) report further argued that even long-term glucocorticoid therapy within recommended ranges may adversely influence cardiac function in children with CAH; its stratified analyses highlight exposure duration and cumulative burden as key explanatory variables to be incorporated into future research designs and follow-up frameworks (35).

#### 2.4.3 Functional reserve and exercise capacity

Direct evidence remains scarce and is highly vulnerable to confounding by underlying disease and co-therapies. In broader pediatric chronic disease populations outside of DMD and CAH, data on cardiopulmonary exercise testing, stress echocardiography, or long-term functional reserve are limited; available findings are often strongly influenced by disease activity and concomitant treatments (e.g., anthracycline chemotherapy or other immunosuppressants), making it difficult to isolate an independent glucocorticoid effect.

#### 2.4.4 Special population

Duchenne muscular dystrophy (DMD) highlights the opposite possibility—potential protection or delayed cardiomyopathy progression. Long-term glucocorticoid use in DMD represents a paradigmatic setting of high cumulative exposure, yet cardiomyopathy progression is also tightly driven by the natural history of the disease. Overall, the clinical literature more consistently supports associations between glucocorticoid therapy and lower all-cause mortality and reduced risk of cardiomyopathy onset/progression, including evidence from observational analyses with propensity adjustment (36). Moreover, a duration–exposure gradient has been described, whereby longer treatment is associated with later onset and/or lower probability of cardiomyopathy (e.g., an approximately 4% reduction in cardiomyopathy probability per additional year of treatment) (37). Cardiac MRI studies further suggest that longer steroid exposure is associated with a lower age-related increase in myocardial fibrosis burden and with more favorable trajectories of LVEF decline (38). Narrative syntheses have consolidated these findings while underscoring persistent heterogeneity in study designs and residual confounding (39). Thus, DMD provides a clinically meaningful boundary condition for interpreting “glucocorticoid–cardiac function” relationships: under specific disease contexts and treatment paradigms, glucocorticoids may confer a net protective effect.

In summary: (1) Across most studies in glucocorticoid-exposed children/adolescents, sustained overt systolic dysfunction is not a consistent finding, and conventional echocardiographic parameters (notably EF) generally remain within normal ranges—suggesting that reliance on traditional measures alone may miss subtle abnormalities (32, 33). (2) However, in long-term exposure settings or selected populations (e.g., CAH), more sensitive assessments (e.g., tissue Doppler indices and MPI) can reveal mild, subclinical systolic–diastolic alterations, with some studies showing dose- or cumulative exposure–related trend signals (35, 36). (3) In DMD, existing evidence suggests that glucocorticoid therapy may delay cardiomyopathy progression and adverse outcomes (36–39). Therefore, conclusions in this field must be interpreted with strict stratification by disease background, exposure intensity (dose/duration/cumulative burden), follow-up window, and the functional assessment technique employed.

### 2.5 Long-term cardiovascular outcomes after childhood glucocorticoid exposure

Overall, direct longitudinal evidence linking glucocorticoid use in childhood/adolescence to “hard” adult endpoints—such as heart failure, arrhythmias, myocardial infarction, or stroke—remains very limited; most population-based studies have instead focused on (1) severe complications occurring during or shortly after treatment (e.g., treatment-emergent hypertension, diabetes, or venous thromboembolism) and (2) subclinical changes that may foreshadow later risk (e.g., endothelial dysfunction or increased carotid intima–media thickness) (40, 42, 43).

Heart failure risk: To date, few cohorts have followed individuals with prolonged/high-dose systemic steroid exposure in childhood into midlife with heart failure as a primary endpoint, precluding any claim that pediatric exposure “necessarily” leads to heart failure. From a risk-pathway perspective, however, oral glucocorticoid use in children shows clear dose- and time-related associations with treatment-emergent hypertension and diabetes—established upstream determinants of future heart failure (40); concurrently, large adult cohorts demonstrate a robust dose–response relationship between oral glucocorticoids and cardiovascular events, including heart failure, with risk signals detectable even at relatively low doses (4). A more defensible interpretation, therefore, is that prolonged and/or higher cumulative exposure in childhood may increase a propensity toward later heart failure by accelerating the accumulation of cardiometabolic risk factors, although confirmation will require substantially longer follow-up windows and direct outcome ascertainment (4, 40).

Arrhythmias: Similarly, pediatric data on the long-term incidence of arrhythmias are sparse, largely due to low baseline event rates, limited follow-up duration, and pronounced confounding by indication. Indirect support comes from adult studies in which glucocorticoid exposure is associated with higher risks of atrial fibrillation and related events in a dose-responsive manner (4). In children and adolescents, if steroid-related hypertension, metabolic derangements, or myocardial remodeling persist over time, a plausible increase in later arrhythmia susceptibility can be hypothesized; at present, however, this should remain explicitly framed as an inferential conclusion rather than an evidence-established outcome (4, 40).

Atherosclerosis and ischemic events: Direct population-level evidence linking childhood steroid exposure to adult coronary disease or stroke also remains insufficient. Nonetheless, in selected pediatric groups that can be viewed as “models” of chronic glucocorticoid exposure—such as CAH requiring long-term replacement—studies have reported impaired vascular function as early as adolescence (e.g., reduced flow-mediated dilation) (43), and some cohorts have observed increased cIMT and treatment duration–related elevations in blood pressure (5). These findings are more consistent with the concept of earlier vascular aging and accelerated subclinical atherosclerosis than with proven increases in adult hard endpoints; whether such signals translate into higher rates of myocardial infarction or stroke will depend on longer-term follow-up with stricter control for confounding (42, 43).

**Figure 3:**
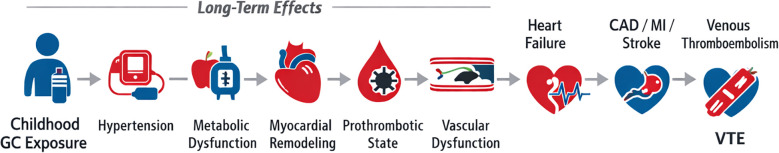
Relationship between childhood glucocorticoid exposure and adult cardiovascular events.

Thrombotic events: Among outcomes that are more directly supported by current data, thrombotic events appear relatively more robust. A large U.S. Medicaid pediatric cohort demonstrated a strong dose- and time-related association between oral glucocorticoids and incident treatment-emergent venous thromboembolism (VTE), characterized by a high relative risk but a low absolute event rate (40); a nationwide adult case–control study similarly linked systemic glucocorticoids to elevated VTE risk (41). Accordingly, when discussing “long-term cardiovascular outcomes,” VTE can reasonably be treated as a representative hard endpoint of steroid-related vascular/coagulation risk, while also underscoring the clinical importance of risk recognition and monitoring (40, 41). Taken together, the currently available evidence supports a conceptual framework in which childhood glucocorticoid exposure contributes to long-term cardiovascular risk primarily through the accumulation of cardiometabolic and vascular alterations, rather than through directly established causal links to hard clinical endpoints; this proposed risk trajectory and its key intermediary pathways are summarized in **Figure 3**.

## 3 Discussion

Overall, direct longitudinal evidence linking systemic glucocorticoid exposure in childhood/adolescence to hard cardiovascular endpoints in adulthood—including heart failure, arrhythmia, myocardial infarction, or stroke—remains scarce. Existing clinical studies tend to converge on two more readily observable domains: (i) severe complications captured during or shortly after treatment (e.g., treatment-related hypertension, diabetes, venous thromboembolism), and (ii) subclinical vascular alterations that may foreshadow longer-term risk (e.g., endothelial dysfunction, increased carotid intima–media thickness) (40, 42, 43).

Heart failure risk. To date, there are no cohort studies that begin with children exposed to long-term/high-dose systemic glucocorticoids, follow them into midlife, and adjudicate heart failure as a primary outcome; accordingly, it is inappropriate to frame childhood exposure as a definitive causal determinant of adult heart failure. From a “risk-chain” perspective, however, pediatric oral glucocorticoid use shows a clear dose-/time-related association with treatment-induced hypertension and diabetes—both well-established antecedents of heart failure later in life (40). In parallel, large adult cohorts consistently demonstrate a dose–response relationship between oral glucocorticoids and cardiovascular events, including heart failure, with excess risk detectable even at relatively low doses (4). A more defensible interpretation is therefore that prolonged or higher cumulative exposure during childhood may increase a propensity toward later heart failure indirectly by accelerating risk-factor accrual, but this hypothesis still requires direct confirmation across longer follow-up windows (4, 40).

Arrhythmia. Evidence on long-term arrhythmia incidence in pediatric populations is similarly limited. Practical barriers include a low baseline event rate, inadequate follow-up duration, and substantial confounding by indication. Indirect support comes from adult studies in which glucocorticoid exposure is associated with a higher risk of atrial fibrillation and related events in a dose-dependent manner (4). In children and adolescents, if glucocorticoid-related elevations in blood pressure, metabolic derangements, or remodeling phenotypes persist, arrhythmia susceptibility might plausibly increase over time; nonetheless, at present, this should be characterized more cautiously as a mechanistic, indirectly supported inference rather than a conclusion grounded in direct pediatric outcome data (4, 40).

Atherosclerosis and ischemic events. Direct population-level evidence connecting childhood glucocorticoid exposure to adult coronary heart disease or stroke also remains insufficient. Yet in selected pediatric populations that can be viewed as “models” of chronic glucocorticoid exposure—most notably long-term replacement therapy in congenital adrenal hyperplasia (CAH)—studies suggest that vascular dysfunction may already be detectable in adolescence (e.g., reduced flow-mediated dilation) (42), and some cohorts report increased carotid intima–media thickness together with treatment-duration–related elevations in blood pressure (5). Collectively, these findings are more consistent with the notion that early vascular aging and subclinical atherosclerosis may emerge earlier. Whether—and under what exposure intensity and disease contexts—these signals translate into higher rates of adult hard endpoints will depend on longer follow-up and more rigorous control of confounding (42, 43).

Thromboembolic events. Among outcomes with comparatively direct support, thromboembolism is backed by more consistent evidence. A large U.S. Medicaid pediatric cohort demonstrated a marked dose-/time-related association between oral glucocorticoids and incident venous thromboembolism (VTE), with increased relative risk despite a low absolute event rate (40). A nationwide adult case–control study similarly links systemic glucocorticoids to higher VTE risk (41). Thus, in discussions of long-term cardiovascular sequelae, VTE can serve as a representative “hard endpoint” for glucocorticoid-associated vascular/coagulation risk, underscoring the clinical need for strengthened risk identification and monitoring in settings of prolonged therapy or high cumulative exposure (40, 41).

To accelerate comparability across pediatric cohorts and reduce avoidable heterogeneity, future studies should adopt a minimal standardized reporting set for systemic glucocorticoid (GC) exposure and cardiac outcomes. For exposure, investigators should report the agent(s), route, indication, start age (developmental window), treatment pattern (continuous vs intermittent/pulse), duration, peak dose, and cumulative burden using a harmonized prednisolone-equivalent metric where feasible. For outcomes, studies should predefine core structural endpoints (e.g., LV mass/LVMI, wall thickness Z-scores, chamber dimensions, CMR-based fibrosis when available) and core functional endpoints (e.g., EF, GLS, tissue Doppler indices, MPI/Tei, E/e′), with consistent timing of assessment. Critically, all analyses should report sex distribution, provide sex-stratified estimates (or sex-by-exposure interaction tests) for each key endpoint, and—where data permit—present dose–response (and/or duration–response) associations using transparent modeling choices. Where clinical events are examined, standardized definitions and adjudication procedures should be reported. Adoption of such a framework would materially improve interpretability and support future pooled analyses and guideline-oriented monitoring strategies.

## 4 Conclusion

A relatively robust conclusion can be drawn from the available evidence: direct and decisive population-level data demonstrating long-term adverse effects of childhood/adolescent glucocorticoid exposure on cardiac structure and function are still lacking; reported structural changes more often present as treatment-related, reversible myocardial hypertrophy, whereas evidence for permanent structural injury remains insufficient (27, 44). Meanwhile, pediatric glucocorticoid exposure shows a clear dose-dependent association with cardiovascular risk factors, including hypertension, dysglycemia, and thrombotic events; these intermediates provide a more plausible mechanistic bridge to the later-life risk of heart failure, coronary disease, and arrhythmias, and they also represent more actionable targets for clinical surveillance and early intervention (4, 40, 41, 45). Therefore, the next critical step is to establish longitudinal, well-phenotyped cohorts that can be followed into adulthood—while minimizing confounding by indication as far as possible—to quantify exposure–response relationships and identify modifiable mediating pathways, thereby moving the field from risk inference toward an evidence base that is measurable, stratifiable, and clinically manageable.
